# Evaluation of Microvessel Density of Hepatocellular Carcinoma and Comparison with Benign Lesions of Liver: An Immunohistochemical Study

**Published:** 2012-02-01

**Authors:** S Amoueian, A Attaranzadeh, F PourAlborzi, M Tarhini, M Montazer

**Affiliations:** 1 Department of Pathology, Mashhad University of Medical Sciences, Mashhad, Iran; 2 Department of Molecular and Cytogenetic Pathology, Shiraz University of Medical Sciences, Shiraz, Iran

**Keywords:** Hepatocellular carcinoma, CD34, Microvessel density, immunohistochemistry

Dear Editor,

Hepatocellular carcinoma (HCC) is one of the most common malignancies in the world[1] that is frequently diagnosed by obtaining core needle biopsies. However, distinguishing a well-differentiated HCC from benign hepatic lesions, such as fatty liver, hepatitis, cirrhosis, and adenomas, particularly in these small specimens, can be quite challenging.[2][3] In this study, we used the microvessel density (MVD) index to help this differentiation.

Samples were consisted of twenty HCCs and seventeen benign hepatic lesions, diagnosed between 1999 and 2009 in the Pathology Department of Ghaem and Imam-Reza hospitals, Mashhad, Iran. To evaluate the MVD, a polymer-based immunohistochemical technique was performed for the detection of CD34, a highly sensitive angiogenesis marker.[4][5] Then, MVD was counted based on the Gasparini's criteria (The Chalkley point).[6] All statistical analyses were carried out using SPSS software. In order to compare means between groups, we used t test or its nonparametric counterpart (Mann-Whitney U test). Receiver Operating Characteristic (ROC) curve analysis was done to assess the diagnostic accuracy of MVD.

The male-to-female ratio was 7:3 for both malignant and benign lesions and the groups were statistically matched for sex. The mean age was higher in patients with malignancy (60.7±15.8 vs. 44.3± 23.2 years old, p=0.024). The only available case of adenomatosis was associated with glycogen storage disease. The mean MVD was higher in HCC than benign lesions (129.1±44.1 vs. 74.0±39.6, p<0.0001). However, there was no statistically significant difference for mean MVD between benign and normal cases (47.8±8.1 vs. 74.0±37.4, p=0.200). Moreover, the mean MVD was not different between two sexes (141.2± 46.1 vs. 124.0±43.9, p=0.440).

Different CD34 staining patterns were also examined. Complete immunostaining pattern was solely found in HCC and in the specimen with hepatic adenoma, while incomplete and negative staining patterns were seen in normal cases, cirrhosis, hepatitis and fatty liver. Immunostaining with sinusoidal pattern around nests and trabecules was found in six HCC cases.

Table 1 shows the diagnostic indices of MVD using a cut-off point of 101 microvessels. In other words, MVD counts of more than 101 vessels are in favor of malignancy; whereas lower counts are seen in benign hepatic lesions.

**Table 1:** Diagnostic indices of microvessel density using CD34 immunostaining

**Parameter**	**Value**	**95% confidence interval **	
		**Upper limit **	**Lower limit **
Sensitivity	0.85	0.94	0.63
Specificity	0.82	0.93	0.58
PPV	0.85	0.94	0.63
NPV	0.82	0.93	0.58
Area Under Curve	0.83	0.96	0.69

Several studies have evaluated the association between CD34 expression and HCC prognosis,[2][7] but only a limited number of investigations have been done to determine the diagnostic value of CD34 immunostaining method.[3] What is more, it has been questioned whether CD34 alone, regardless of its staining pattern, can be used to distinguish benign liver nodules from HCC. Based on a research done by Tanigava. et al., anti-CD34 staining was confined to vessels of the portal triad with weak staining in few sinusoids at the periportal area in benign lesions such as cirrhosis or chronic hepatitis cases (mean MVD± SD, 23±5 /0.74 mm2). However, intensive and specific staining of sinusoid-like vessels was observed in all of the tumoral lesions using anti-CD34 antibody (mean MVD± SD, 297±88 /0.74 mm2).[8]

Although MVD can be evaluated by several markers such as VEGF, CD105, vWF and CD34, some researchers have claimed that CD34 is the most sensitive marker.[2][8] We also achieved good results with high sensitivity using this marker.

Overall, it seems that the MVD in HCC is higher compared to benign hepatic lesions except in cases of adenoma and FNH. Furthermore, the complete CD34 staining pattern was virtually always found in HCC, and rarely in its benign mimickers. Conclusively, the results of this study show that the evaluation of MVD-CD34 in association with its immunostaining pattern, contributes to a reliable distinction between HCC and non-neoplastic liver diseases.
